# Mechanistic Insights into Archaeal and Human Argonaute Substrate Binding and Cleavage Properties

**DOI:** 10.1371/journal.pone.0164695

**Published:** 2016-10-14

**Authors:** Sarah Willkomm, Adrian Zander, Dina Grohmann, Tobias Restle

**Affiliations:** 1 Institute of Molecular Medicine, Universitätsklinikum Schleswig-Holstein, Universität zu Lübeck, Lübeck, 23538, Germany; 2 Department of Biochemistry, Genetics and Microbiology, Institute of Microbiology- Archaea Centre, University of Regensburg, Regensburg, 93053, Germany; National University of Singapore, SINGAPORE

## Abstract

Argonaute (Ago) proteins from all three domains of life are key players in processes that specifically regulate cellular nucleic acid levels. Some of these Ago proteins, among them human Argonaute2 (hAgo2) and Ago from the archaeal organism *Methanocaldococcus jannaschii* (MjAgo), are able to cleave nucleic acid target strands that are recognised via an Ago-associated complementary guide strand. Here we present an in-depth kinetic side-by-side analysis of hAgo2 and MjAgo guide and target substrate binding as well as target strand cleavage, which enabled us to disclose similarities and differences in the mechanistic pathways as a function of the chemical nature of the substrate. Testing all possible guide-target combinations (i.e. RNA/RNA, RNA/DNA, DNA/RNA and DNA/DNA) with both Ago variants we demonstrate that the molecular mechanism of substrate association is highly conserved among archaeal-eukaryotic Argonautes. Furthermore, we show that hAgo2 binds RNA and DNA guide strands in the same fashion. On the other hand, despite striking homology between the two Ago variants, MjAgo cannot orientate guide RNA substrates in a way that allows interaction with the target DNA in a cleavage-compatible orientation.

## Introduction

Argonaute (Ago) proteins are found in all three domains of life [1]. In eukaryotes, Ago proteins are involved in a posttranscriptional gene silencing process called RNA interference (RNAi) [2]. Besides translational inhibition, Ago-mediated cleavage reduces target mRNA levels in a sequence-specific way [2,3]. In humans, of the four Ago variants only hAgo2 is capable of target RNA cleavage [4,5]. The sequence-specificity of this process is ensured by Ago-bound short interfering RNAs (siRNA) of 21–25 nt length generated from long double-stranded (ds) precursor RNAs [6–8]. Processing of the precursor RNAs is mediated by Dicer, an RNase-III-like enzyme [8,9]. Within the RNA-induced silencing complex (RISC)-loading complex (RLC) consisting of Dicer, hAgo2 and a double-stranded (ds) RNA binding protein—either trans-activation response (TAR) RNA-binding protein (TRBP) or protein activator of protein kinase R (PKR) (PACT)—the mature siRNA is loaded into hAgo2 [10–15]. One of the strands, the so-called guide strand, remains bound to hAgo2 whereas the other strand, termed passenger strand, is cleaved and eventually ejected [16–18] leaving behind RISC [19], minimally consisting of hAgo2 and a guide strand [20]. Based on complementarity to a hAgo2-bound guide strand, target RNAs are recognised by RISC and are subsequently subject to hAgo2-mediated RNA cleavage [21]. As a multiple turnover enzyme RISC is able to perform multiple rounds of cleavage [22–24].

Ago proteins consist of four different domains, which are arranged in a bilobal fashion [25–32]. The catalytic activity is harboured by the PIWI domain, which displays an RNaseH–like fold [22,25,29,33,34]. Together with the Mid domain, the PIWI domain forms the C-terminal lobe of Ago. During the formation of binary Ago-guide complexes the Mid domain binds the 5’-end of the guide strand [27,28,35]. The N-terminal lobe is composed of the PAZ (Piwi-Argonaute-Zwille) and the N-terminal domain. The 3’-end of the guide strand is initially bound by the PAZ domain [36] and released upon binding of a matching target RNA to form catalytically active ternary Ago-guide-target complexes [20,21,37,38]. The N-terminal domain seems to contain important features for the cleavage activity of Ago [39,40] and is involved in duplex unwinding [41].

Recently, we provided detailed mechanistic insights into binary and ternary complex formation for hAgo2 and developed a minimal mechanistic model of siRNA-dependent hAgo2-mediated target RNA cleavage [20,42]. Among others, this model provides information about the velocity of the different transitions during guide and target RNA binding, which makes it a valuable tool for comparative analyses of different guide and target substrates and/or different Ago proteins.

Despite striking structural homology between eukaryotic and prokaryotic Agos [28], proteins from different domains of life display remarkable differences [43]. In contrast to eukaryotic Agos, the physiological function of prokaryotic Agos is not well understood. Recent studies revealed that bacterial Agos might be involved in defence against foreign genetic material [44,45]. While human Agos predominantly use RNA as guide and target substrates, some bacterial Agos have been shown to utilize both DNA and RNA [21,27,32,34,44–47]. Among the prokaryotic Agos characterized so far, Ago from the archaeal organism *Methanocaldococcus jannaschii* (MjAgo) belongs to a group of Ago proteins that are exclusively cleaving DNA substrates [37], albeit, binding studies disclosed ternary protein-nucleic acid complexes with RNA targets are formed.

In this study we carried out a detailed analysis of DNA and RNA substrate binding kinetics and the corresponding cleavage process. Comparing MjAgo and hAgo2 side-by-side we were able to reveal the mechanisms that govern substrate binding and cleavage by MjAgo. Additionally, we shed light on the conservation of mechanistic pathways throughout evolution. Furthermore, first evidence on how different Ago proteins discriminate between DNA and RNA substrates is provided.

## Results

### MjAgo and hAgo2 bind corresponding guide strands in a comparable way

**Fig 1:**
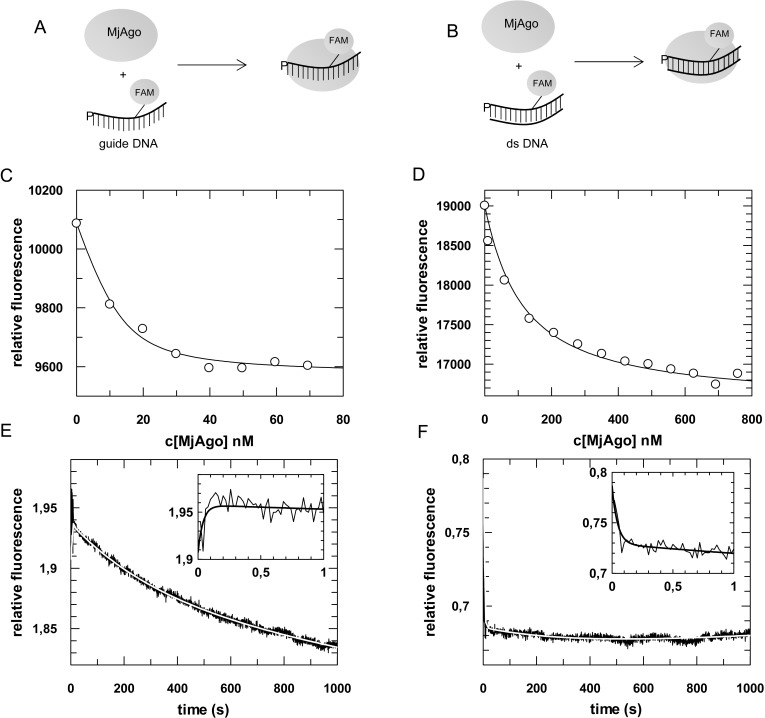
Formation of binary MjAgo-nucleic acid complexes. Binding of either fluorescently labelled (A) ss guide DNA or (B) ds siDNA to MjAgo was monitored under (C, D) equilibrium or (E, F) pre-steady state conditions. (C) ss guide DNA (D-as2b^FAM^, 20 nM) or (D) ds siDNA (D-si2b^FAM^, 20 nM) were titrated with increasing concentrations of MjAgo. Both data sets were mathematically evaluated using a quadratic equation. The best fit of the experimental data to a quadratic equation is shown for representative measurements. The fit yielded *K*_*D*_ values of 3.1 (± 0.9) nM and 102 (± 6.6) nM for the binary interaction of MjAgo with ss guide DNA and ds siDNA. Next, binary complex formation was analysed by rapidly mixing (E) 600 nM or (F) 700 nM MjAgo with (E) 20 nM ss guide DNA (D-as2b^FAM^) or (F) ds siDNA (D-si2b^FAM^). Representative graphs are shown. The inset shows the data on a shorter time scale. Data were fitted best using a triple exponential equation, yielding the following rate constants: (E) *k*_1_obs_: 25. 0 (± 6.2) s^-1^, *k*_2_: 0.23 (± 0.02) s^-1^ and *k*_3_: 0.003 (± 0.0002) s^-1^ and (F) *k*_1_obs_: 22.1 (± 1.5) s^-1^, *k*_2_: 0.24 (± 0.005) s^-1^ and *k*_3_: 0.003 (± 0.0002) s^-1^.

Formation of binary MjAgo-guide complexes was analysed analogous to previously described binding studies with hAgo2 [20]. To allow a direct comparison of the two studies the same oligonucleotide sequence was used and the fluorophore was placed at position 14 of the guide DNA (Table 1). At first we analysed MjAgo binding to ss guide DNA (D-as2b^FAM^) and the corresponding ds siDNA (D-as2b^FAM^/D-s2b) under equilibrium conditions (Fig 1C and 1D), yielding *K*_D_ values of 3.4 (± 0.4) and 103 (± 1.5) nM, respectively. Hence, binding affinities of MjAgo for a ss DNA guide strand or a ds siDNA are comparable to the affinities determined for hAgo2 and corresponding RNA substrates (Table 2; [20]).

**Table 1:** Sequences of oligonucleotides used in this study.

name	sequence (5’-3’)
**as2b**	uag agg uac gug cug agg cTT
**as2b**^**FAM**^	uag agg uac gug cT*g agg cTT
**D-as2b**	TAG AGG TAC GTG CTG AGG CTT
**D-as2b**^**FAM**^	TAG AGG TAC GTG CT*G AGG CTT
**D-as2b**^**14FAM_21Cy5**^	TAG AGG TAC GTG CT*G AGG CTT^#^
**s2b**	gcc uca gca cgu acc ucu aTT
**s2b**^**FAM**^	gcc uca gca cgu acc ucu aTT*
**D-s2b**	GCC TCA GCA CGT ACC TCT ATT
**D-s2b**^**mm**^	GCC TCA GCA GCG GGA ATA TTT

Capital letters represent deoxynucleotides. Oligonucleotides used as a guide strand were 5’-phosphorylated.

*position of the C6-linked 5/6-FAM and

^#^position of Cy5.

**Table 2:** Overview of equilibrium and pre-steady state binding data of MjAgo and hAgo2 binary complex formation.

	*k*_1_bin_ (M^-1^ s^-1^)	*k*_2_bin_ (s^-1^)	*k*_3_bin_ (s^-1^)	*K*_D_bin_ (nM)
	*collision complex*	*5’-end Mid binding*	*3’-end PAZ binding*	
**MjAgo**				
**guide**^**DNA**^	0.3 (±0.005) x 10^8^	0.3 (±0.1)	0.005 (±0.003)	3.4 (±0.4)
**ds siDNA**	0.3 (±0.005) x 10^8^	0.23 (±0.02)	0.003 (±0.002)	103 (±1.5)
**guide**^**DNA**^**w/ bulky 3’-label**	n.d.	0.021 (±0.006)	—	n.d.
**MjAgo Mid**^**mut**^				
**guide**^**DNA**^	n.d.	—	0.002 (±0.0001)	n.d.
**hAgo2**				
guide^RNA^*	0.6 (±0.001) x 10^8^	0.26 (±0.02)	0.012 (±0.0005)	7 (±0.9)
ds siRNA*	1.2 (±0.13) x 10^8^	0.48 (±0.23)	0.028 (±0.018)	48 (±7)
guide^DNA^	0.1 (±0.0009) x 10^8^	0.1 (±0.005)	0.003 (±0.001)	n.d.

*data taken from Deerberg et al. [20]. *K*_D_bin_ is the average of at least two independent equilibrium titrations. n.d.: not determined. The rate constants *k*_2_bin_ and *k*_3_bin_ are averaged from at least three independent measurements. Standard deviations in brackets. For *k*_1_ standard errors are given.

Next, pre-steady state analyses were performed with a stopped-flow device using the same fluorescently labelled substrates (ss guide and ds siDNA) mentioned above. Fluorescently labelled substrates were rapidly mixed with different concentrations of MjAgo. In both cases data obtained were mathematically evaluated using a triple exponential equation (Fig 1E and 1F) revealing that the first fast phase was dependent on the protein concentration (Fig Ai and Aii in S1 File) whereas the subsequent slower phases were not. Evaluation of the concentration dependency of the first phase employing a linear fit yielded a second-order rate constant of 0.3 (± 0.005) x 10^8^ M^-1^ s^-1^ for both substrates with corresponding back rates of 6.6 (± 3.3) and 4.1 (± 2.4) s^-1^ for ss guide DNA and ds siDNA, respectively. The rate constants of the succeeding slower phase determined with ss guide or ds siDNA are nearly identical to the corresponding rates determined for hAgo2 and ss guide RNAs or ds siRNAs (Table 2). Accordingly, we propose that this phase represents binding of the guide’s 5’-end to the MjAgo Mid domain analogous to the mechanism described for hAgo2 [20]. Pre-steady state experiments using a MjAgo Mid mutant further supported this postulate. Here, the amino acid tyrosine 442 which corresponds to tyrosine 529 in hAgo2 was mutated to an alanine (Fig L in S1 File). For hAgo2 this position was shown to be important for guide 5’-end binding [28,30,35,48]. Moreover, Ma et al. [32] showed that already a point mutation in the guide RNA 5’-end binding region severely disturbs formation of functionally active RISC. Accordingly, we expected reduced Mid binding for this MjAgo mutant as well. In fact, stopped-flow experiments revealed a two-step kinetic (Fig B in S1 File) instead of the three-step process observed with wildtype MjAgo (Fig 1E). Here, the second transition is missing which clearly supports the notion that this phase indeed corresponds to Mid binding of the guide 5’-end. Moreover, the rate constant of the remaining slow phase is in good agreement with the third phase determined with wildtype MjAgo implying that the guide strand is attached to the PAZ domain. Such a scenario is supported by TtAgo X-ray crystal structures in complex with a 10-mer guide [19] and demonstrates that even though a guide strand is not bound to the Mid domain it still can interact with the PAZ binding pocket.

Further support for our postulation that the three phases of MjAgo guide binding can be assigned to the same structural transitions as in case of hAgo2 [20] comes from pre-steady state experiments with MjAgo and a guide substrate carrying a bulky fluorophore at the 3’-end. In earlier studies [37] employing electromobility shift assays (EMSA) with MjAgo and a guide strand with a bulky 3’-label we could show that a voluminous addition to the guide’s 3’-end prevents binary complex formation. This underlines the importance of PAZ binding for efficient formation of binary MjAgo-guide complexes. On the other hand, since detection of target binding is still possible, most probably due to altered interactions between MjAgo and nucleic acids in the ternary complex [37], we conclude, even though the interaction of the guide 3’-end to the PAZ domain is heavily disturbed, the 5’-seed region is still properly positioned. Congruent with these results, when employing the stopped-flow technique we found that a guide DNA with a bulky 3’-fluorophore is bound in a two-step process (Fig C in S1 File). Since PAZ binding is most likely blocked by the bulky fluorophore our results imply that the second phase observed represents Mid binding. Interestingly, the rate constant does not match the one determined with the canonical guide strand. It is slowed down by one order of magnitude which strongly indicates that guide 3’-end PAZ binding is also important for a coordinated association of the guide strand 5’-region within the Ago nucleic acid binding channel.

**Fig 2:**
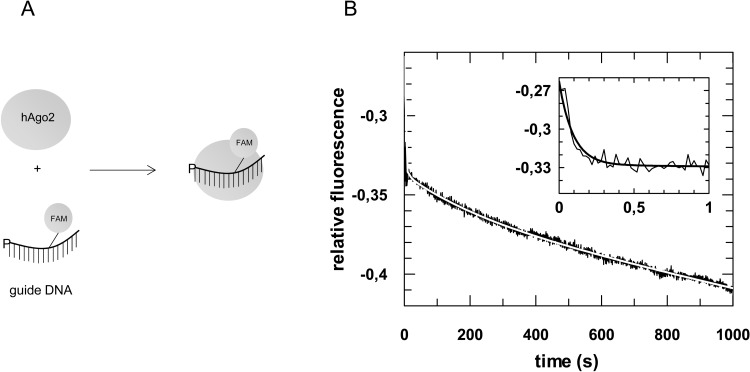
Pre-steady state kinetics of binary hAgo2-guide^DNA^ complex formation. (A) Schematic representation of the conducted experiment. (B) Binary complex formation was analysed by rapidly mixing 500 nM hAgo2 with 20 nM ss guide DNA (D-as2b^FAM^). A representative graph is shown. The inset shows the data on a shorter time scale. Data were fitted best using a triple exponential equation yielding the following rate constants: *k*_1_obs_: 10.6 (± 0.4) s^-1^, *k*_2_: 0.11 (± 0.01) s^-1^ and *k*_3_: 0.004 (± 0.0003) s^-1^.

In contrast to the corresponding phase determined for hAgo2 and RNA guide substrates, the third phase of MjAgo binary complex formation is significantly slower for single stranded and—even more pronounced—for double-stranded DNA substrates. This is consistent with the assignment of this rate constant to PAZ binding with the MjAgo Mid mutant (Fig B in S1 File). For binary hAgo2-guide complex formation the third phase was found to represent 3’-end binding of the guide to the PAZ domain [20]. In order to determine whether 3’-end binding of the guide by hAgo2 also occurs with DNA guide strands, we analysed binary complex formation of hAgo2 and ss guide DNA (D-as2b^FAM^) instead of ss guide RNA (Fig 2). We found that hAgo2 binding to DNA guides follows a three-step process as well. Likewise, the first fast phase is dependent on the protein concentration (Fig D in S1 File), whereas the second and third phases are not. Interestingly, the third phase is almost identical to the third phase of binary complex formation determined with MjAgo and a DNA guide strand (Table 2). As guide 3'-end binding to the PAZ domain was shown to contribute to MjAgo binary complex formation [37], we conclude that the observed third phase of complex formation between Ago and a guide strand represents this interaction and propose this process is generally slower with DNA as compared to RNA guides. In addition, the experiments with hAgo2 and guide DNA revealed that hAgo2 binds DNA guides in a similar fashion as cognate RNA substrates enabling correct target strand binding as supported by cleavage assays performed earlier by Lima et al. [49]. In contrast, MjAgo presumably is not able to bind an RNA guide in the same orientation as a DNA guide. It does not use RNA guides to guide cleavage of a target strand [37] and therefore we were not able to detect formation of ternary complexes composed of MjAgo, RNA guides and DNA targets (data not shown).

### MjAgo ternary complexes are formed with DNA as well as RNA target strands

Next, we analysed the formation of ternary MjAgo-guide-target complexes. As observed for hAgo2 [20], steady state fluorescence titrations revealed that the affinity of binary MjAgo-guide DNA complexes for target DNA (Fig E in S1 File) is very similar to the affinity of two isolated nucleic acid strands (i.e. guide-target hybrid) in absence of protein [20].

**Fig 3:**
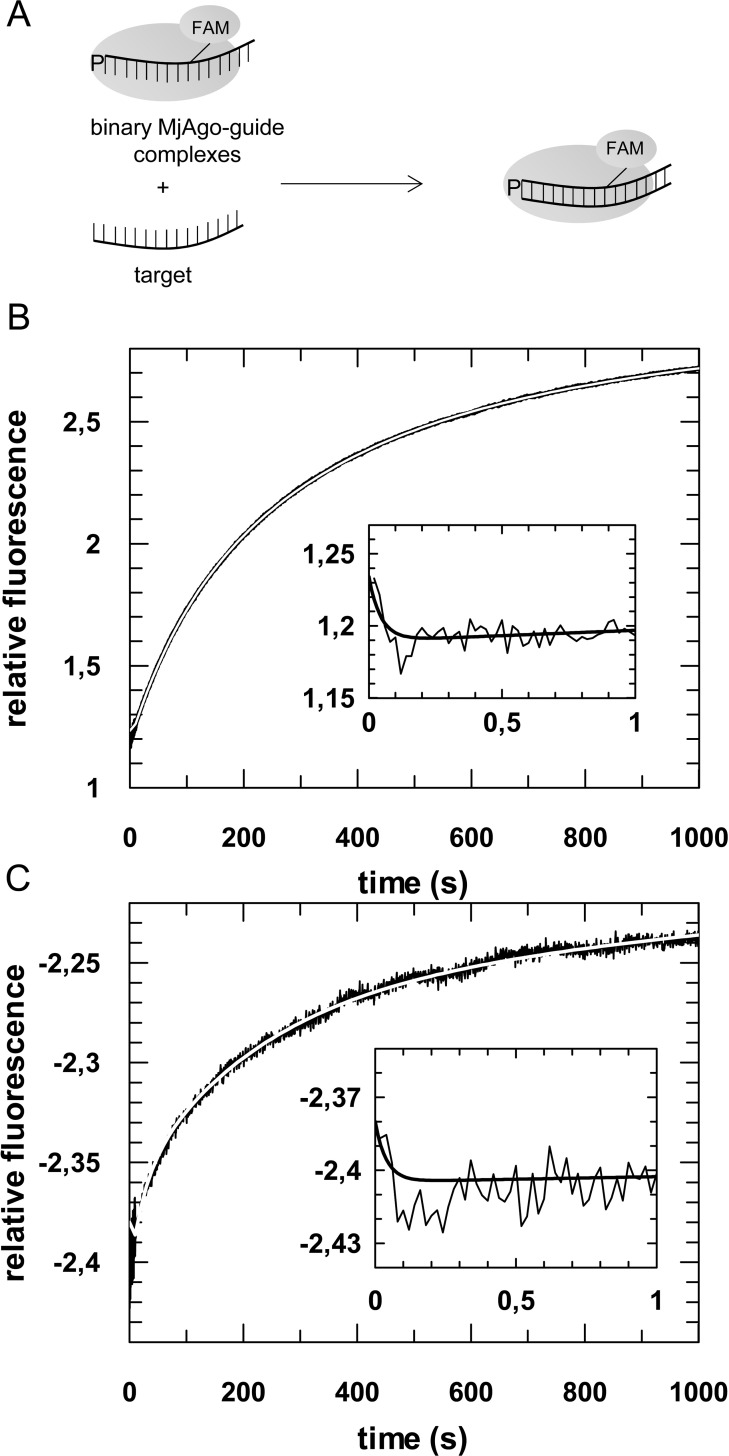
Association of ternary MjAgo-guide-target complexes. (A) Schematic representation of the experimental setup; MjAgo is pre-assembled with a fluorescently labelled DNA guide and subsequently mixed with an unlabelled guide-matching target (DNA or RNA) strand. Pre-assembled binary complexes consisting of 500 nM MjAgo and 20 nM guide DNA (D-as2b^FAM^) are rapidly mixed with either (B) 20 nM target DNA (D-s2b) or (C) 40 nM target RNA (s2b). Representative graphs are shown. The insets show the data on a shorter time scale. Data could be fitted best using a triple exponential equation, yielding the following rate constants: (B) *k*_1_obs_: 21.8 (± 3.0) s^-1^, *k*_2_: 0.01 (± 0.0003) s^-1^ and *k*_3_: 0.003 (± 0.00007) s^-1^ or (C) *k*_1_obs_: 25.1 (± 10.5) s^-1^, *k*_2_: 0.04 (± 0.0005) s^-1^ and *k*_3_: 0.004 (± 0.00001) s^-1^.

Pre-steady state analyses revealed that analogous to hAgo2, MjAgo ternary complex formation is a three-step process with rate constants being very close to those determined for the human enzyme (Fig 3 and Table 3). Taking into consideration that the structures of prokaryotic and human Agos are highly related [28,43], it is plausible to suggest that the target binding phases observed with binary MjAgo-guide complexes most probably correspond to the same structural transitions described for hAgo2 [20]. Again, the first fast phase is dependent on the concentration of the binding partners (Fig F in S1 File) and represents the formation of collision complexes between binary MjAgo-guide complexes and target DNA. We propose the subsequent phase represents base pairing between the seed region of the guide DNA and the target DNA. This conclusion is subsidized by pre-steady state analyses using a target being complementary to the guide strand in the seed region only (Fig G in S1 File). In contrast to a fully complementary target the seed-matched target DNA is bound by the binary MjAgo-guide complex in a two-step process. A comparison to the kinetics determined for the binding of fully complementary target reveals that a third phase is undetectable and the second phase closely matches the second phase determined with a fully complementary target strand supporting the interpretation that this phase of ternary complex formation corresponds to base-pairing in the seed region. Subsequently, the 3’-end of the guide DNA is released from the PAZ domain [37] which allows extended base pairing between guide and target strand which is most probably reflected by the third phase of ternary complex formation. In contrast, association of guide and target in absence of MjAgo followed a two-step process with a diffusion-limited first phase followed by a slower phase. This second step is about 3-fold slower compared to the protein-assisted association of guide and target in the seed region (Table 3) indicating that MjAgo facilitates Watson-Crick base pairing between the nucleic acid strands. This is most probably due to a pre-arranged seed region that does not require conformational changes to bind a target strand as observed in crystal structures of binary complexes with other Ago proteins [28–30,19,50]. A close inspection of the different phases discloses that neither different substrates nor slight structural differences between the two Agos have an effect on the kinetics of ternary complex formation (Table 3). A difference between ternary complexes with hAgo2 and MjAgo and their cognate substrates can only be found regarding dissociation rate constants. For MjAgo dissociation of guide and target strand in the seed region is faster as compared to hAgo2. This might be due to weaker interactions within DNA-DNA hybrids as compared to RNA-RNA hybrids [51]. Interestingly, if hAgo2 is bound to RNA-DNA or DNA-RNA hybrids dissociation within the seed region is likewise increased (Table 3) supporting the assumption that interactions between guide-target duplexes and Ago are dependent on the nature of the nucleic acids. Analogous to hAgo2 [20] the rate-limiting step for MjAgo ternary complex dissociation (*k*_-3-ter_) likely corresponds to dissociation of the target strand from the 3’-region of the guide strand.

**Table 3:** Overview of equilibrium and pre-steady state binding data of MjAgo and hAgo2 ternary complex formation.

	*k*_1_ter_ (M^-1^ s^-1^)	*k*_-1_ter_ (s^-1^)	*k*_2_ter_ (s^-1^)	*k*_-2_ter_ (s^-1^)	*k*_3_ter_ (s^-1^)	*k*_-3_ter_ (s^-1^)	*K*_D_ter_ (nM)
	*collision complex*		*seed pairing*		*PAZ release and 3’-base pairing*		
**MjAgo**							
**guide**^**DNA**^ **target**^**DNA**^	1.2 (±0.007) x 10^8^	6.3 (±2.4)	0.014 (±0.004)	0.012 (±0.0003)	0.0042 (±0.0017)	0.0003 (±0.00005)	0.5 (±0.04)
**guide**^**DNA**^ **target**^**RNA**^	n.d.	n.d.	0.04 (±0.02)	n.d.	0.0033 (±0.0006)	n.d.	n.d.
**guide**^**DNA**^**target**^**3’mmDNA**^	n.d.	n.d.	0.018 (±0.001)	n.d.	—	—	n.d.
**hAgo2**							
**guide**^**RNA**^ **target**^**DNA**^	2.6 (±0.2) x 10^8^	1.2 (±0.6)^#^	0.005 (±0.002)	0.03 (±0.004)	0.0007 (±0.0001)	0.0003 (±0.0001)	0.6 (±0.2)
**guide**^**DNA**^ **target**^**RNA**^	3.5 (±0.003) x 10^8^	0.9 (±0.07)^#^	0.005 (±0.0003)	0.02 (±0.007)	0.002 (±0.0004)	0.0004 (±0.00005)	0.8 (±0.1)
**guide**^RNA^* target^**RNA**^	3.2 (±0.4) x 10^8^	11.8 (±2.0)	0.01 (±0.002)	0.003 (±0.0018)	0.003 (±0.0009)	0.0002 (±0.0001)	0.2 (±0.04)
**w/o Ago**							
**DNA/DNA**	n.d	n.d	0.003 (±0.0003)	n.d	—	—	

*data taken from Deerberg et al. [20].

^#^rate constants taken from the ordinate intercept of the linear fit of the concentration dependency of *k*_1_obs_. *K*_D_ter_ is the average of at least two independent equilibrium titrations. n.d.: not determined. The rate constants *k*_2_ter_ and *k*_3_ter_ are averaged from at least three independent measurements. Standard deviations in brackets. For *k*_1_ and *k*_-1_ standard errors are given.

**Fig 4:**
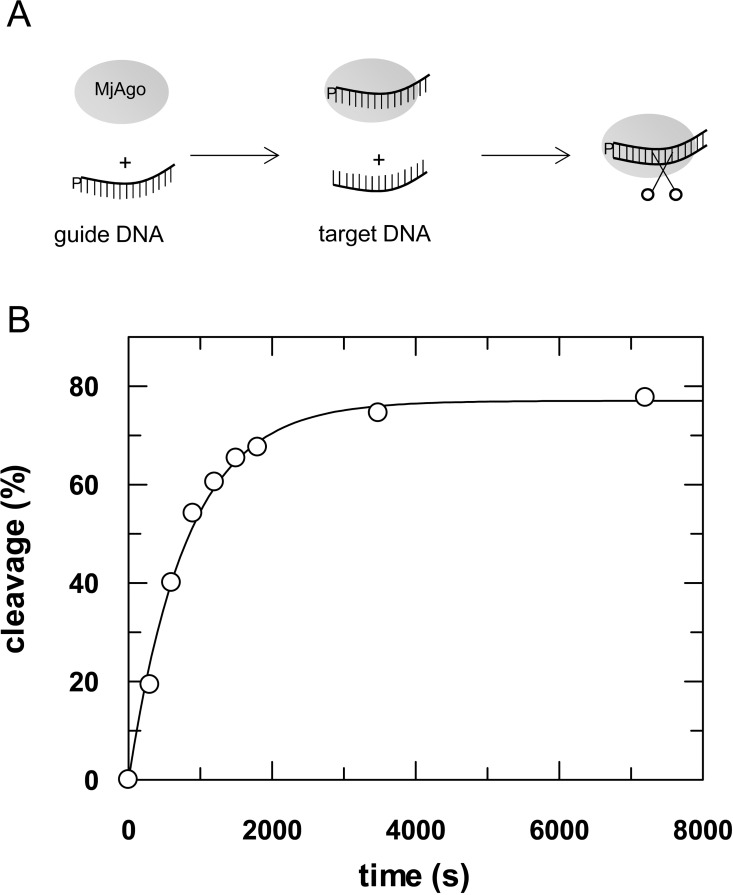
MjAgo-mediated target DNA cleavage under single turnover conditions. (A) Schematic representation of the experimental setup. MjAgo was premixed with guide DNA. Cleavage was started by adding a radioactively labelled DNA target strand. Cleavage experiments were conducted using 1 μM MjAgo, 100 nM guide strand (D-as2b) and 2.5 nM target strand (^32^P-D-s2b) at 85°C. Samples were taken at time points 0’, 5’, 10’, 15’, 20’, 25’, 30’, 60’ and 120’, separated on 20% denaturing polyacrylamide gel and visualized by autoradiography. Relative cleavage amplitudes were plotted versus time. Data could be fitted best using a single exponential equation yielding a rate constant *k*_*cleavage*_: 0.0012 (± 0.00007) s^-1^. The average of three independent measurements yielded a rate constant of 0.0014 (± 0.0003) s^-1^.

In a next step, we analysed cleavage mediated by MjAgo using its cognate substrates. To this end, we preassembled binary MjAgo-guide DNA complexes and started the reaction by adding target DNA. Under single turnover conditions, MjAgo cleaves its target with a rate constant of 0.0014 (± 0.0003) s^-1^ (Fig 4). This rate constant is in the same range as the rate constant determined for association of guide and target DNA bound to MjAgo beyond the seed region (Table 3). Base pairing in the central and 3’-base region is very important for efficient target cleavage [52]. Turnover under the given conditions is therefore limited by the velocity of base pairing between guide and target in the central and 3’-region.

Previously conducted cleavage assays with MjAgo and RNA instead of DNA targets demonstrated that MjAgo is not able to cleave an RNA target [37]. However, binding studies disclosed ternary complex formation when RNA targets were used. We therefore wondered whether a detailed analysis of the kinetics of this process would reveal a mechanistic explanation for this finding. Pre-steady state kinetic analyses showed ternary complex formation is not affected by target strand chemistry, with rate constants for the different steps being nearly identical for DNA and RNA targets (Fig 3A and 3C and Table 3). These findings indicate that there might be an additional structural transition necessary to adopt a conformation that allows target strand cleavage, which cannot be monitored in the assays conducted in the context of this study. This transition might be inhibited by a non-cognate target.

### hAgo2 cleavage kinetics with DNA guides are equivalent to RNA guides

**Fig 5:**
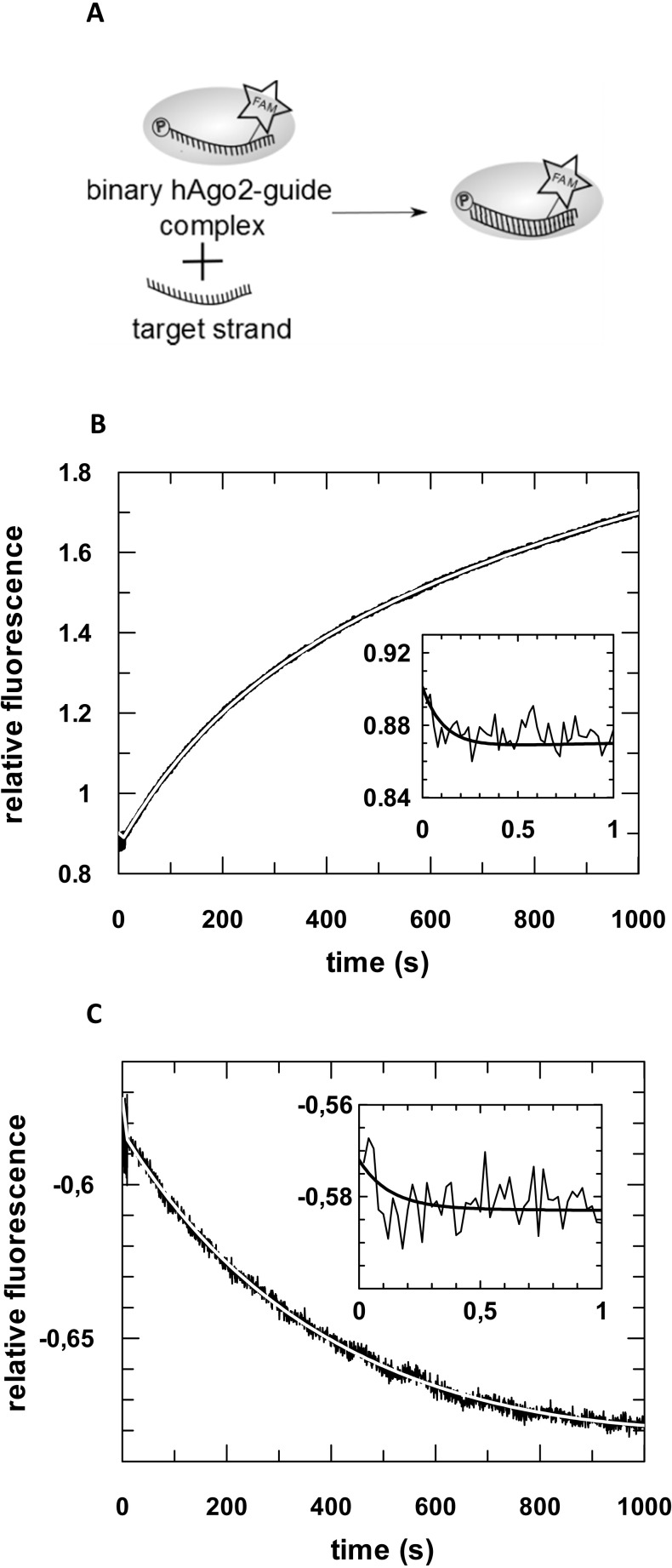
Association of different ternary hAgo2-guide-target complexes. (A) Schematic representation of the experimental setup; binary complexes composed of hAgo2 and a fluorescently labelled guide strand (RNA or DNA) are preassembled and subsequently mixed with a matching target strand (DNA or RNA). Preassembled binary complexes consisting of 500 nM hAgo2 and 20 nM (B) guide RNA (as2b^FAM^) or (C) guide DNA (D-as2b) are rapidly mixed with 20 nM (B) target DNA (D-s2b) or (C) target RNA (s2b^FAM^). Representative graphs are shown. The inset shows the data on a shorter time scale. Data could be fitted best using a triple exponential equation, yielding the following rate constants: (B) *k*_1_obs_: 9.2 (± 0.9) s^-1^, *k*_2_: 0.005 (± 0.00009) s^-1^ and *k*_3_: 0.0007 (± 0.00004) s^-1^ and (C) *k*_1_obs_: 7.9 (± 0.8) s^-1^, *k*_2_: 0.004 (± 0.0004) s^-1^ and *k*_3_: 0.001 (± 0.0001) s^-1^.

In contrast to MjAgo, hAgo2 is capable of forming functional binary complexes with DNA as well as RNA guides (Table 2). As *in vivo* eukaryotic Argonautes are thought to be exclusively loaded with small RNAs [44,45], we wondered whether a mechanistic difference in ternary complex formation with DNA and RNA guides can be found (Fig 5 and Table 3). Hence, we tested DNA versus RNA targets.

**Fig 6:**
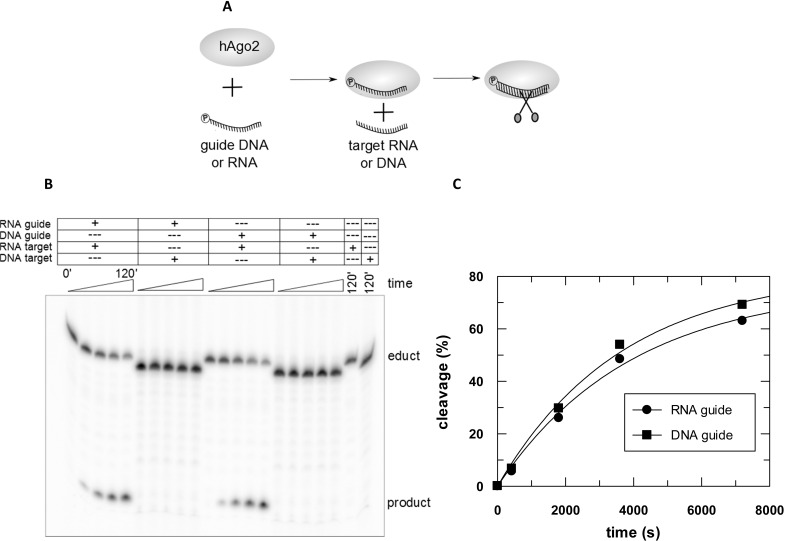
hAgo2 cleavage assay with RNA or DNA targets guided by RNA or DNA. (A) Cleavage experiments were conducted using 2.5 μM hAgo2, 100 nM guide strand and 2.5 nM target strand at 37°C. Samples were taken at time points 0’, 10’, 30’, 60’ and 120’, separated on 20% denaturing polyacrylamide gel and visualised by autoradiography. (B) Bands were evaluated using ImageQuant 5 and relative cleavage amplitudes were plotted versus time. (C) Data could be fitted best with a single exponential equation yielding a rate constant *k*_*cleavage*_: 0.0003 s^-1^ for RNA cleavage irrespective of the guide strand chemistry. The average of two independent measurements yields a rate constant of 0.0003 (± 0.00007) s^-1^ in both cases. In case of DNA targets no cleavage could be observed.

Equilibrium titrations of binary hAgo2-guide RNA (as2b^FAM^) and hAgo2-guide DNA (D-as2b^FAM^) complexes with target DNA (D-s2b) or target RNA (s2b) revealed *K*_D_ values of 0.6 (± 0.2) and 0.8 (± 0.1) nM, respectively (Fig I in S1 File). Compared to ternary hAgo2-guide RNA-target RNA complexes [20], the affinity of ternary complexes with DNA/RNA or RNA/DNA guide-target duplexes are lower (Table 3). With respect to the affinity of these complexes, it seems to be of no relevance whether the DNA proportion represents the guide or the target strand, though. Performing pre-steady state analyses of ternary complex formation we were able to uncover differences between ternary complexes with different guide-target combinations (Fig 5 and Fig J in S1 File). For the rate constant of the second phase of ternary complex association it did not matter whether DNA or RNA was used as guide or rather target strand (Table 3). However, the rate constant of the third phase of ternary complex formation is different. This phase represents guide 3’-end release from the PAZ domain, associated structural changes and extended base pairing of guide and target strand in the 3’-region of the guide [20]. Accordingly, interactions of the target strand with hAgo2 might induce structural transitions which lead to the release of the guide 3’-end from the PAZ domain. While the rate constant for this transition with RNA/RNA or DNA/RNA guide-target combination are highly similar, it is significantly slowed down in case of RNA/DNA guide-target (Table 3). Thus, aforementioned target-induced guide 3'-end PAZ dissociation might be impaired in case of a DNA target. Additionally, this implies a deviant positioning of the DNA target within hAgo2-guide-target complexes supported by the results of cleavage experiments which revealed that DNA targets are no subject to hAgo2-mediated cleavage neither with an RNA nor with a DNA guide (Fig 6). A DNA target might be positioned too far away from the hAgo2 active site to allow cleavage.

While association kinetics of ternary complexes with different guide-target combinations did not show differences, corresponding dissociation kinetics (Fig K in S1 File) revealed a significant change in the rate constant of the second phase with DNA/RNA or RNA/DNA guide-target combinations (Table 3). This phase represents dissociation of guide and target substrate within the seed region [20] leading us to the postulation that the interactions in this region are weaker in case of a DNA/RNA hybrid compared to a RNA/RNA hybrid.

Cleavage assays conducted with hAgo2 and different guide-target combinations provided further insight into the nucleic acid binding properties of hAgo2. As previously shown by Lima et al. [49], hAgo2 is able to cleave RNA targets using DNA as well as RNA guide strands (Fig 6). Kinetic analyses of target RNA cleavage by hAgo2 guided by RNA or DNA demonstrate that under multiple turnover conditions the nature of the guide substrate seems to be irrelevant (Fig 6C). The cleavage rate constants are congruent implying that in both cases the cleavage reaction is limited by dissociation of cleavage products.

## Discussion

**Fig 7:**
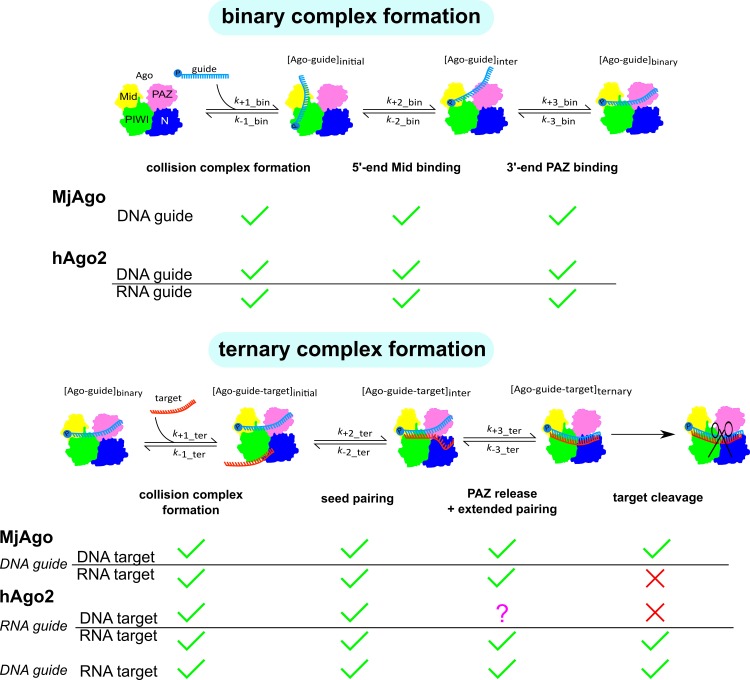
Schematic representation of the nucleic acid binding properties of hAgo2 and MjAgo. Ago is represented in cartoons based on X-ray structures with N-, PAZ, Mid and PIWI domain coloured individually (N-terminal domain: blue; PAZ: magenta; Mid: yellow; PIWI: green). Relative positions of Ago, guide and target strand are indicated. The corresponding rate constants for each step are given in Table 2. Green checkmarks indicate the individual step is taking place, red crosses show that the indicated step could not be observed. The question mark indicates that this transition could not be definitely assigned.

One intention of this work was to directly compare substrate binding kinetics of an archaeal Ago to those of hAgo2 based on a previously established minimal mechanistic model of siRNA-dependent hAgo2-mediated target RNA cleavage [20]. In-depth steady state and pre-steady state kinetic studies of MjAgo guide and target binding were conducted and revealed a striking overall similarity on the mechanistic level. However, this study also disclosed important differences between hAgo2 and MjAgo substrate binding mechanisms. To further explore those differences we additionally studied substrate tolerance of both proteins for different RNA and DNA guide-target combinations (summarised in Fig 7).

Pre-steady state analyses of MjAgo guide binding revealed a three step process as shown before for hAgo2 [20]. On the basis of largely congruent rate constants and extensive structural homology between prokaryotic and eukaryotic Ago proteins [1,28] we concluded these steps most likely correspond to the same structural transitions as shown for the human enzyme. A detailed analysis of the Mid domains of a hAgo2 X-ray crystal structure and a MjAgo homology model [37] revealing a striking homology between both proteins supports this conclusion. Residues that have been shown earlier to be important for 5’-nucleotide binding of the guide strand by the Mid domain [32,35] are also highly conserved in MjAgo (Fig Li in S1 File). Even though the homology model of the MjAgo PAZ domain and the X-ray crystal structure of hAgo2 display less similarity (Fig Lii in S1 File), single-molecule FRET studies showed that the guide 3’-end is anchored in the MjAgo PAZ domain [37]. Accordingly, we propose that the first phase of MjAgo binary complex formation represents a diffusion-limited collision between MjAgo and the guide DNA, followed by anchoring of the guide’s 5’-end in the Mid binding pocket and subsequent binding of the guide’s 3’-end to the PAZ domain (illustrated in Fig 7). In comparison to hAgo2, it turned out that the third phase of MjAgo binary complex formation is significantly slower (Table 2). This also holds true for ds siDNA. Analysing binary complex formation with hAgo2 and a guide DNA instead of a guide RNA revealed this being a substrate chemistry-dependent phenomenon as the third phase in the association process between hAgo2 and a DNA guide instead of a RNA guide is slowed down as well. Structural studies show that the 3'-end of the guide strand is threaded through a narrow channel between the hAgo2 N-terminal and PAZ domain [31]. The measured third rate constant of ternary complex formation might at least in part reflect this structural transition which is certainly strongly affected by the chemical nature of the substrate (i.e. RNA versus DNA). Thus, we conclude the 2’-OH group plays an important role to facilitate structural changes that allow guide binding to the N-terminal part of the nucleic acid binding channel. This idea is supported by X-ray data, which demonstrated that especially 2’-OH groups of the sugars in the 5’-region of the guide strand are interacting with hAgo2 residues [28,30].

Earlier studies showed MjAgo does not slice targets when bound to an RNA guide and the affinity for such a guide is strongly reduced [37]. This finding suggests that a guide RNA cannot be correctly positioned within the enzyme. While hAgo2 is able to use DNA guides to cleave RNA targets [49], MjAgo does only cleave targets when bound to a DNA [37]. The additional 2’-OH groups of the guide RNA presumably require extra space which is interfering with binding to MjAgo. These different substrate tolerance mechanisms of hAgo2 and MjAgo suggest a difference in evolution. While for hAgo2 there seems to be no need to distinguish between different guide substrates, its prokaryotic homologue is restricted to DNA guide strands. While hAgo2 probably mainly finds small RNA and no short DNA substrates in the cytoplasm, prokaryotic Agos might have to differentiate between different guide substrates. Besides their natural DNA guides, RNA substrates can potentially interact with MjAgo in the cytosol. In archaea, numerous small non-coding RNAs have been identified [53–56] that are for example substrates for the prokaryotic RNA chaperone Hfq [55], which can theoretically also be bound by prokaryotic Ago.

Further kinetic analyses revealed a very similar behaviour of hAgo2 and MjAgo at the step of ternary complex formation. Steady state measurements demonstrate that binding of a short target strand is primarily mediated via Watson-Crick base pairing and both bind to their cognate targets in a three-step process with rate constants for the different transitions being nearly identical (Table 3). It is likely, in consequence of a less tight interaction between DNA guide and DNA or RNA target strands in comparison to RNA/RNA hybrids, that the rate representing dissociation of the strands within the seed region is slightly faster in case of MjAgo. The rate constant of the third phase of ternary complex dissociation is nearly identical between MjAgo and hAgo2, indicating that the substrate being RNA or DNA is not influencing this transition. Moreover, this finding implies that the structural transitions necessary to eject a bound target or cleavage product are the same for the two enzymes. Thus, dissociation of cleavage products [20–22] being the rate limiting step of target cleavage seems to be a universal concept of Ago-mediated gene regulation.

Interestingly, despite the differences observed concerning guide substrate preference both hAgo2 and MjAgo are able to bind RNA as well as DNA targets. However, the rate constants reflecting different transitions during ternary complex formation disclose differences between the archaeal and mammalian enzymes. While MjAgo is binding and releasing RNA targets with the same rate constants as DNA targets, hAgo2 shows target-specific differences. Using RNA/DNA hybrid substrates, irrespective which one is guide or target strand, dissociation of the two strands within the seed region was observed to be significantly faster. This implies that interactions of hAgo2 with a guide-target duplex depend on the chemical nature of the substrate duplex, which in turn affects the strengths of the intermolecular nucleic acid interaction. Crystal structures of hAgo2 in complex with a guide and a short target RNA revealed that hAgo2 is stabilising the A-form guide-target duplex by an extensive network of interactions between residues of helix-7 and the minor groove of the duplex [31]. While the minor groove architecture of RNA/RNA duplexes is highly comparable to that of RNA/DNA hybrids, the major groove of RNA/DNA hybrids is much larger than in the RNA/RNA duplexes leading to an average helix conformation that is in-between A- and B-form [57]. Possibly, this causes clashes of the target strand with helix-7 and would explain the observed increase in the rate constants of dissociation in the seed region. Moreover, such a proposed destabilisation is reflected by elevated *K*_d_’s for ternary complexes with RNA/DNA hybrids. Even though archaeal Ago proteins apparently possess a helix comparable to hAgo helix-7 [30] our results do not provide any evidence that MjAgo behaves like hAgo2 when interacting with RNA/DNA hybrids. For the prokaryotic *T*.*thermophilus* Ago it was discovered that guide-target duplexes composed of either only DNA or DNA and RNA both adopt an A-form like helix conformation [21] despite the fact that DNA duplexes naturally adopt a B-form like conformation. In combination with the kinetic data presented here this suggests that prokaryotic Ago proteins might be able to shape the conformation of the guide-target helix, whereas hAgo2 is not.

Despite the fact that ternary complex stability is affected, hAgo2-DNA guide complexes cleave RNA targets at the same speed as compared to hAgos loaded with an RNA guide implying the actual cleavage step is much faster than the observed cleavage rate constant of 0.0003 s^-1^ [20]. If instead of an RNA target a DNA target is used to assemble ternary hAgo2-guide-target complexes, the third phase of ternary complex association is remarkably slower. Together with the finding that hAgo2 is not cleaving DNA targets we conclude that a DNA target is not properly aligned within the ternary complex, at least with respect to the 3’-region of the guide strand, which in turn might eventually affect active site geometry. In contrast to hAgo2 we did not observe any differences in the rate constants when ternary MjAgo-substrate complexes were formed with either DNA or RNA targets. On the other hand, MjAgo does not cleave RNA targets. In order to resolve these contradicting observations we propose a fourth transition during ternary complex assembly, which cannot be observed in our experiments. The transition most likely represents conformational rearrangements leading to a catalytically active ternary complex. One reason why such a phase is missing in the experiments presented is that the proposed conformational change is temperature-dependent. Earlier studies disclosed that MjAgo-mediated cleavage is only possible at temperatures above 75°C [37]. For technical reasons it was impracticable to measure pre-steady state kinetics at high temperatures. An alternative explanation is that the structural transition required for cleavage is very small and cannot be detected by the applied fluorescence-based read out.

In summary, our data demonstrate that the structural homology between eukaryotic and prokaryotic Agos [28] is also reflected in an overall mechanistic similarity. However, a detailed analysis using steady state and especially pre-steady state methods revealed interesting differences in the guide substrate tolerance of eukaryotic hAgo2 and prokaryotic MjAgo. For example, as opposed to MjAgo, there seems to be no necessity for hAgo2 to discriminate between different guide substrate chemistries. On the other hand, when it comes to target cleavage both enzymes strictly select for cognate substrate chemistry underscoring the importance of individual target recognition mechanisms. Data presented here furthermore demonstrate that pre-steady state analyses of Ago-dependent silencing processes based on the previously developed minimal mechanistic model of siRNA-dependent hAgo2-mediated target RNA slicing [20] is well suited to shed light on differences between Ago proteins on the mechanistic level.

## Materials and Methods

### Protein Expression and Purification

MjAgo and hAgo2 were purified and expressed as described before [20,37].

### Oligonucleotides

Unmodified and fluorescein amidite (FAM) labelled RNA and DNA oligonucleotides were obtained PAGE-purified from IBA (Göttingen) or MWG Eurofins (Munich). The sequences of labelled and unlabelled oligonucleotides used in this study are listed in Table 1. Guide strands were either ordered in a 5’-phosphorylated form or phosphorylated using unlabelled ATP (Fermentas). For cleavage assays, target strands were 5’-phosphorylated using [γ-^32^P] ATP (Perkin and Elmer). Phosphorylated oligonucleotides were purified using Sephadex G50 columns (GE Healthcare). Ds DNAs were generated by incubating equimolar concentrations of guide and target strands in hybridization buffer (15 mM Hepes, pH 7.4, 50 mM KCH_3_COOH and 1 mM MgCH_3_COOH) for 5 min at 95°C, and subsequently slowly cooled down to 37°C. Success of hybridization was tested using 20% native PAGE followed by visualization via the FAM label.

### Equilibrium fluorescence titrations

Affinities of hAgo2 or MjAgo for guide and target substrates were measured in hAgo2 binding buffer (10 mM Tris pH 7.5, 100 mM KCl) or MjAgo binding buffer (10 mM Tris pH 7.5, 100 mM KCl and 1 mM MgCl_2_) using a 700 μl quartz cuvette at a constant temperature of 25°C. Either 20 nM of FAM-labelled guide strand or binary complexes (500 nM of one of the Ago proteins and 20 nM of a FAM-labelled guide strand) were titrated with increasing concentrations of Ago or target strand, respectively. The fluorophore was excited at 492 nm and the change of fluorescence upon binding was recorded at 516 nm with slits set to 1 nm using a Fluoromax-3 spectrometer (Horiba Jobin Yvon). Data were mathematically evaluated with GraFit 5.2 (Erithacus Software) using a quadratic equation (Fluorescence = Fmax − ((c[guide] + L + Kd) − sqrt((sqr(c[guide] + L + Kd)) − 4*c[guide]*L))*(Fmax − Fmin)/((2*c[guide])), where Fmax is the maximum fluorescence, Fmin is the minimum fluorescence, c[guide] is the concentration of substrate, L is the concentration of the titration partner, and Kd is the equilibrium dissociation constant).

### Pre-steady state stopped-flow experiments

Binding experiments under pre-steady state conditions were performed using the same binding buffers as listed above at a constant temperature of 25°C. 20 nM FAM-labelled guide substrate was rapidly mixed with different concentrations of one of the Ago proteins to analyse binary complex formation. To monitor ternary complex formation, binary complexes of Ago and FAM-labelled guide strand were preassembled and rapidly mixed with different concentrations of unlabelled target substrate. In some cases instead of the guide, the target substrate was labelled (for details see corresponding figure legends). Dissociation of binary or ternary complexes was measured by rapidly mixing preformed FAM-labelled guide strand containing complexes with an excess of unlabelled competitor nucleic acid. Information on competitor nucleic acids as well as individual concentrations of binding partners can be found in the corresponding figure legends. Change of fluorescence over time was recorded using the Stopped-Flow SX 20 device (Applied Photophysics). Data were fitted using GraFit 5.2 by employing exponential equations of the form Fluorescence = ∑A_n_*exp (−k_n_*t), where A_n_ is the amplitude corresponding to the observed phase, k_n_ is the rate constant of the observed phase, and t is the time.

### Target cleavage assay

Each Ago protein was incubated with guide and radio-labelled target strand (for concentrations see figure legends) in Ago cleavage buffer (10 mM Tris pH 7.5, 100 mM KCl and 2 mM MgCl_2_) with 0.25 mg/ml tRNA and if appropriate 10μg/ml RiboLock RNase Inhibitor (Fermentas). Cleavage reactions with hAgo2 or MjAgo were conducted at 37° or 85°C, respectively. Samples were taken at different time points and the reaction was stopped by addition of 1 volume stop buffer (95% formamide, 0.025% (w/v) SDS, 0.025% (w/v) bromophenol blue, 0.025% (w/v) xylene cyanol, 0.5 mM EDTA). Samples were analysed using denaturing PAGE and visualized by autoradiography.

## Supporting Information

S1 File File includes Figs A–J. Fig A. Concentration dependencies of the first phase of ss guide DNA and ds siDNA binding by MjAgo (observed pseudo-first order rate constant) are shown, Fig B. Kinetics of the association of the MjAgo binding deficient Mid mutant to guide DNA, Fig C. Kinetics of the association of MjAgo with a guide DNA carrying a bulky 3’-label, Fig D. Concentration dependency of the first phase of binary complex assembly with hAgo2 and guide DNA (observed pseudo-first order rate constant), Fig E. Equilibrium titration of binary MjAgo-guide DNA complexes with guide DNA, Fig F. Concentration dependency of the first phase of binary MjAgo-guide DNA complexes binding to target DNA (observed pseudo-first order rate constant), Fig G. Kinetics of ternary complex assembly with MjAgo and a 3’-mismatched target DNA, Fig H. Kinetics of the dissociation of ternary MjAgo/guide/target complexes, Fig I. Equilibrium titrations of binary hAgo2-guide RNA or hAgo2-DNA guide complexes with target DNA or RNA, Fig J. Concentration dependencies of the first phases of ternary complex formation with hAgo2 and guide DNA or RNA with RNA or DNA targets (observed pseudo-first order rate constants), Fig K. Kinetics of ternary complex dissociation of hAgo2-RNA guide-DNA target and hAgo2-DNA guide-RNA target complexes, Fig L. Structural alignment of hAgo2 and MjAgo Mid and PAZ domains.
(PDF)
