# Supplementary material for: Mechanistic Insights into Archaeal and Human Argonaute Substrate Binding and Cleavage Properties
Source: PLoS One. 2016 Oct 14;11(10):e0164695. doi: 10.1371/journal.pone.0164695 (PMC5065165; doi:10.1371/journal.pone.0164695)
Supplement: S1 File — File includes Figs A–J. Fig A. Concentration dependencies of the first phase of ss guide DNA and ds siDNA binding by MjAgo (observed pseudo-first order rate constant) are shown, Fig B. Kinetics of the association of the MjAgo binding deficient Mid mutant to guide DNA, Fig C. Kinetics of the association of MjAgo with a guide DNA carrying a bulky 3’-label, Fig D. Concentration dependency of the first phase of binary complex assembly with hAgo2 and guide DNA (observed pseudo-first order rate constant), Fig E. Equilibrium titration of binary MjAgo-guide DNA complexes with guide DNA, Fig F. Concentration dependency of the first phase of binary MjAgo-guide DNA complexes binding to target DNA (observed pseudo-first order rate constant), Fig G. Kinetics of ternary complex assembly with MjAgo and a 3’-mismatched target DNA, Fig H. Kinetics of the dissociation of ternary MjAgo/guide/target complexes, Fig I. Equilibrium titrations of binary hAgo2-guide RNA or hAgo2-DNA guide complexes with target DNA or RNA, Fig J. Concentration dependencies of the first phases of ternary complex formation with hAgo2 and guide DNA or RNA with RNA or DNA targets (observed pseudo-first order rate constants), Fig K. Kinetics of ternary complex dissociation of hAgo2-RNA guide-DNA target and hAgo2-DNA guide-RNA target complexes, Fig L. Structural alignment of hAgo2 and MjAgo Mid and PAZ domains. (PDF) [file pone.0164695.s001.pdf]

<u>**Supplementary Information**</u>

Mechanistic Insights into Archaeal and Human Argonaute Substrate Binding and Cleavage Properties

Sarah Willkomm[1], Adrian Zander[2], Dina Grohmann[2] and Tobias Restle[1]

[1]Institute of Molecular Medicine, Universitätsklinikum Schleswig-Holstein, University of Lübeck, Lübeck, 23538, Germany

[2]Department of Biochemistry, Genetics and Microbiology, Institute of Microbiology- Archaea Centre, University of Regensburg, Regensburg, 93053, Germany

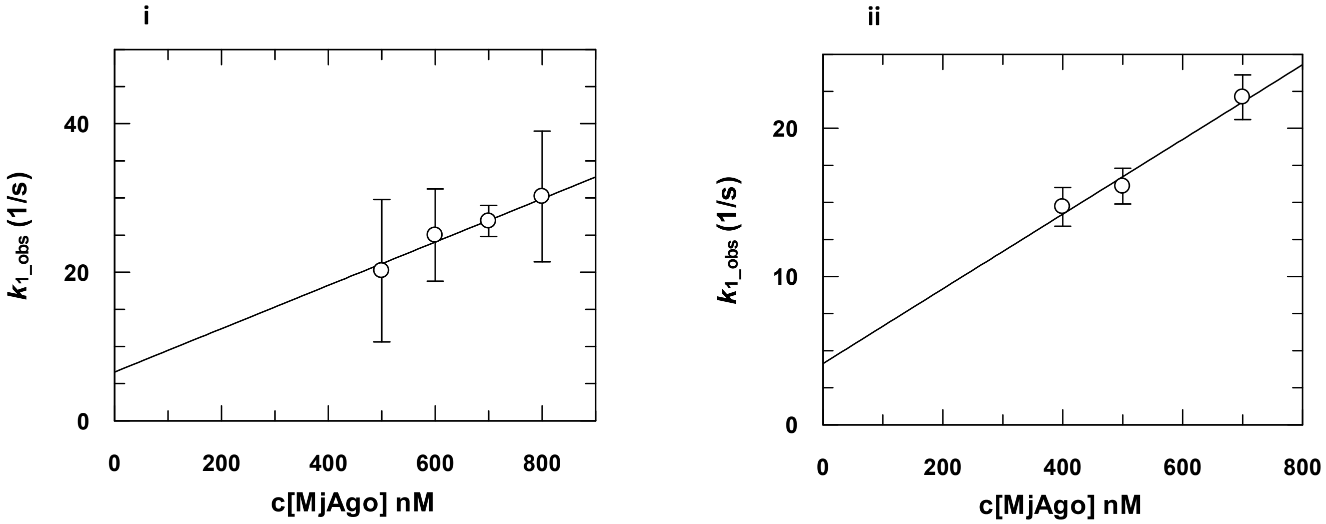

Supplementary Figure A: Linear regression analyses of the concentration dependency of the first phase of binary MjAgo-DNA complex formation. Linear regression analysis of the concentration dependency of the observed first phase k_{1_obs} (see Figure 1 E and F) using a constant concentration of substrate and increasing concentrations of MjAgo yielded the rate constants (i) k_1: 0.3 (± 0.005) x 10^8 M^{-1} s^{-1} and k_{-1}: 6.6 (± 3.3) s^{-1} for binary complex formation with ss guide DNA and (ii) k_1: 0.3 (± 0.005) x 10^8 M^{-1} s^{-1} and k_{-1}: 4.1 (± 2.4) s^{-1} for binary complex formation with ds siDNA.

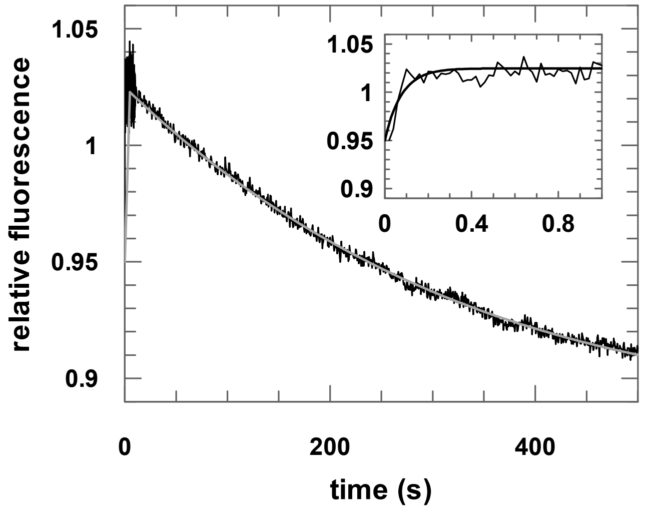

Supplementary Figure B: Pre-steady state kinetics of complex formation between MjAgo-Tyr442Ala and a DNA guide strand. Complex formation was analyzed by rapidly mixing 500 nM MjAgo-Tyr442Ala with 20 nM ss guide DNA (D-as2b^{FAM}). A representative graph is shown. The inset shows the data on a shorter time scale. Data were fitted best using a double exponential equation, yielding the following rate constants: k_{1_obs}: 12.9 (± 0.7) s^{-1} and k_2: 0.0022 (± 0.00005) s^{-1}.

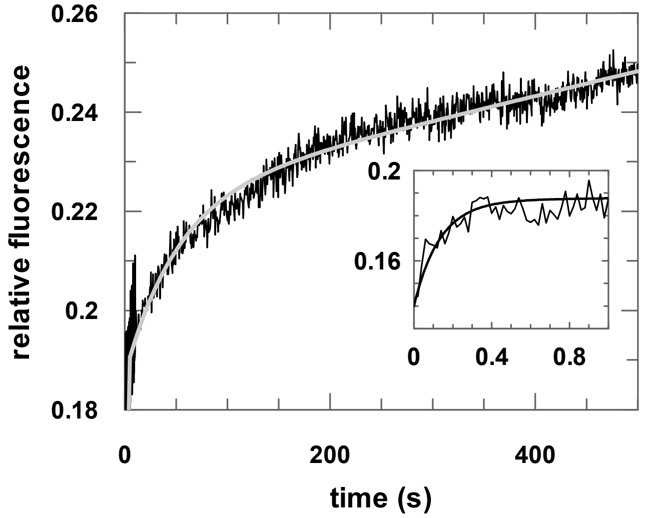

Supplementary Figure C: Pre-steady state kinetics of complex formation between MjAgo and a DNA guide strand with a bulky 3'-label. Complex formation was analyzed by rapidly mixing 300 nM MjAgo with 20 nM ss guide DNA (D-as2b^{14FAM_21Cy5}). A representative graph is shown. The inset shows the data on a shorter time scale. Data were fitted best using a double exponential equation, yielding the following rate constants: k_{1_obs}: 7.5 (± 0.5) s^{-1} and k_2: 0.02 (± 0.0008) s^{-1}.

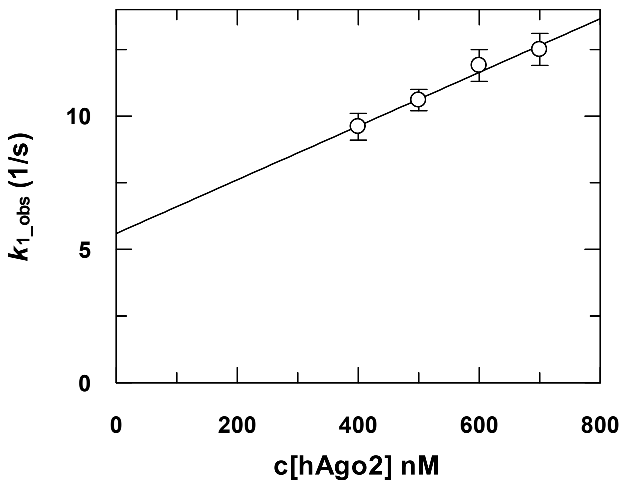

Supplementary Figure D: Linear regression analyses of the concentration dependency of the first phase of binary hAgo2-guide DNA complex formation. Linear regression analysis of the concentration dependency of the observed first phase (see Figure 2) yielded the rate constants k_1: 0.1 (± 0.0009) x 10^8 M^{-1} s^{-1} and k_{-1}: 5.6 (± 0.5) s^{-1}.

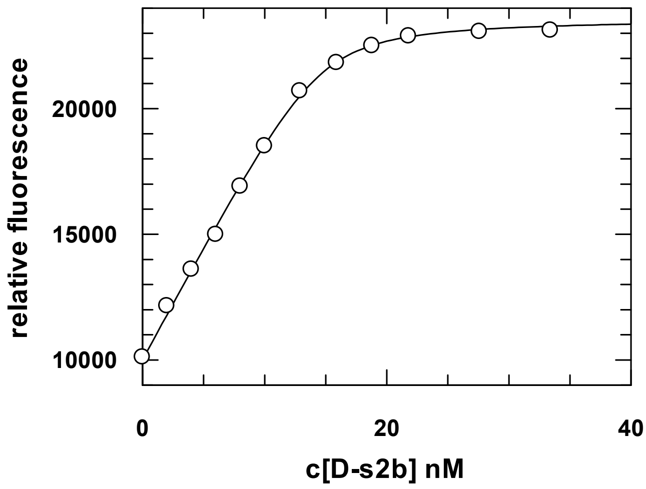

Supplementary Figure E: Equilibrium titrations of binary MjAgo-guide DNA complexes with target DNA. Guide DNA (D-as2b^{FAM}, 20 nM) was preassembled with 500 nM MjAgo and titrated with increasing concentrations of D-s2b. Data were mathematically evaluated using a quadratic equation. The best fit of the experimental data to a quadratic equation is shown for a representative experiment. The fit yielded a K_D of 0.5 (± 0.07) nM for ternary MjAgo-guide-target complexes.

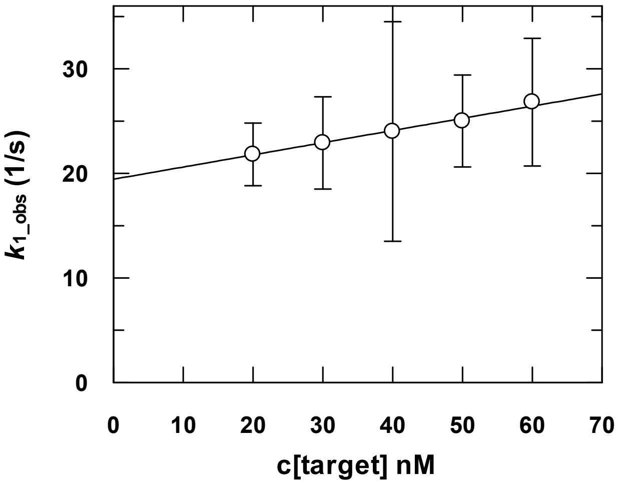

Supplementary Figure F: Linear regression analyses of the concentration dependency of the first phase of ternary MjAgo-guide DNA-target DNA complex formation. Linear regression analysis of the concentration dependency of the observed first phase (see Figure 3 B) yielded the rate constants k_1: 1.2 (± 0.007) x 10^8 M^{-1} s^{-1} and k_{-1}: 19.4 (± 0.2) s^{-1}.

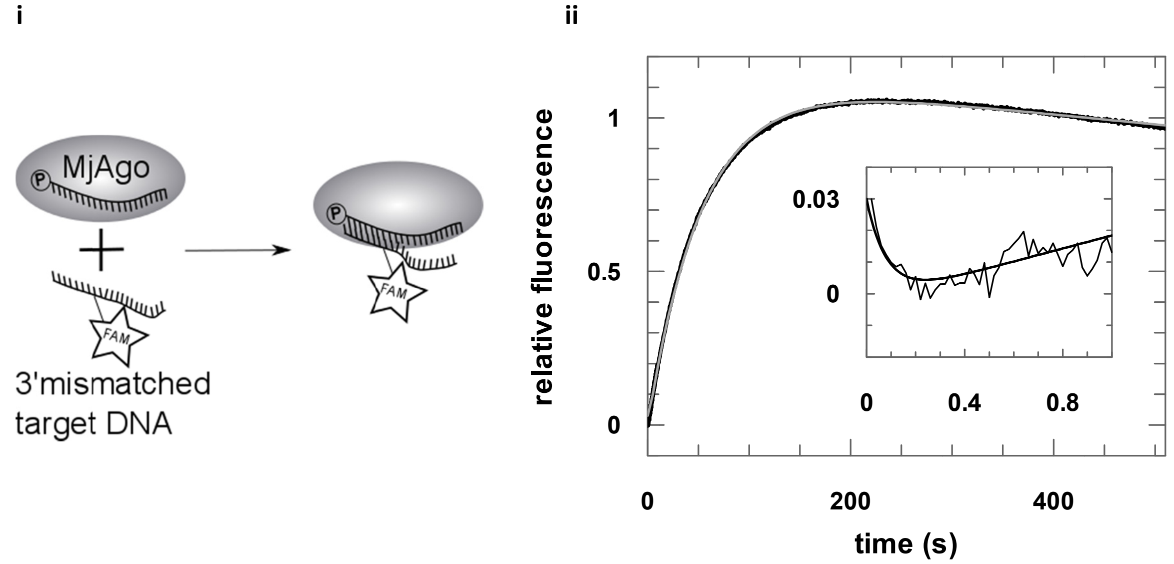

Supplementary Figure G: Association of ternary MjAgo-guide-target complexes with a 3'-mismatched target DNA. (i) Schematic representation of the experimental setup; MjAgo is pre-assembled with a DNA guide and subsequently mixed with a fluorescently labelled 3'-mismatched target strand. Pre-assembled binary complexes consisting of 500 nM MjAgo and 20 nM guide DNA (D-s2b^{mm}) are rapidly mixed with 60 nM target DNA (OH-D-as2b^{FAM}). A representative graph is shown. The insets show the data on a shorter time scale. Data could be fitted best using a double exponential equation, yielding the following rate constants: k_{1_obs}: 12.3 (± 1.9) s^{-1} and k_2: 0.019 (± 0.00003) s^{-1}.

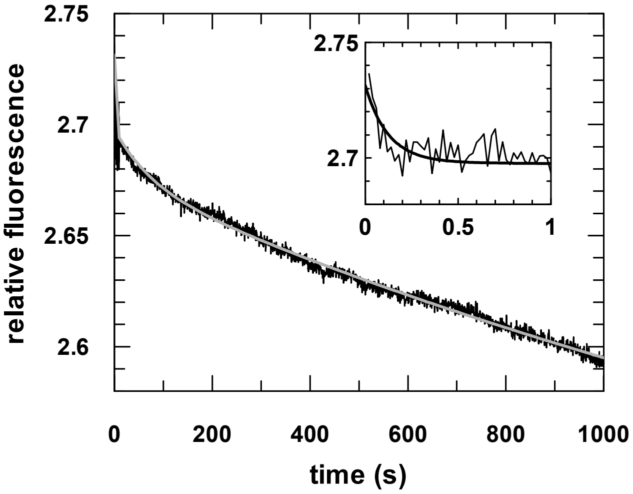

Supplementary Figure H: Dissociation of ternary MjAgo-guide-target complexes in presence of guide competitor excess. Preassembled ternary complexes consisting of 400 nM MjAgo, 20 nM guide DNA (D-as2b^{FAM}) and 40 nM target DNA (D-s2b) were rapidly mixed with unlabeled guide competitor DNA. A representative graph is shown. The inset shows the reaction on a shorter time scale. Data could be fitted best using a triple exponential equation yielding the following rate constants k_{-1}: 8.0 (± 0.8) s^{-1}, k_{-2}: 0.01 (± 0.0006) s^{-1} and k_{-3}: 0.0003 (± 0.00003) s^{-1}.

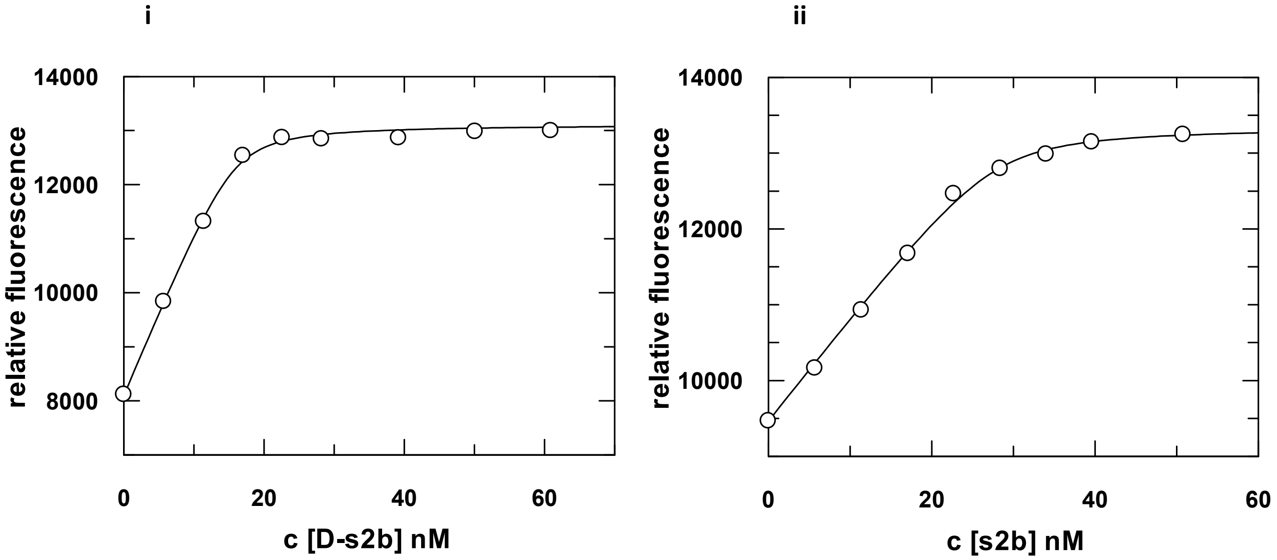

Supplementary Figure I: Equilibrium titrations of binary hAgo2-RNA or hAgo2-DNA complexes with DNA or RNA target. (i) Guide RNA (as2b^{FAM}, 20 nM) or (ii) guide DNA (D-as2b^{FAM}, 20 nM) was preincubated with 500 nM hAgo2 and subsequently titrated with increasing concentrations of D-s2b or s2b. Data were mathematically evaluated using a quadratic equation. The best fit of the experimental data to a quadratic equation is shown for representative measurements. The fits yielded K_D*'s* of (i) 0.5 (± 0.1) nM and (ii) 0.7 (± 0.2) nM, respectively.

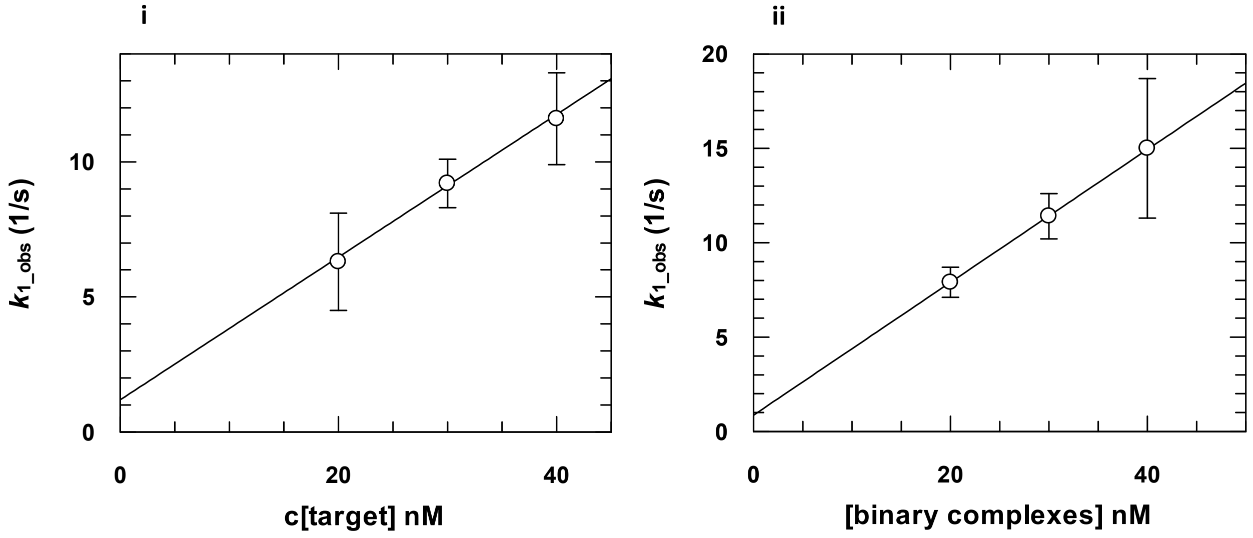

Supplementary Figure J: Linear regression analyses of the concentration dependency of the first phase of different ternary hAgo2-guide-target complex formation. Linear regression analysis of the concentration dependency of the observed first phase (see Figure 5 B and C) yielded the rate constants (i): k_1: 2.6 (± 0.2) x 10^8 M^{-1} s^{-1} and k_{-1}: 1.2 (± 0.6) s^{-1} for ternary hAgo2-guide RNA- target DNA complexes and (ii): k_1: 3.5 (± 0.003) x 10^8 M^{-1} s^{-1} and k_{-1}: 0.86 (± 0.07) s^{-1} for ternary hAgo2-guide DNA-target RNA complexes

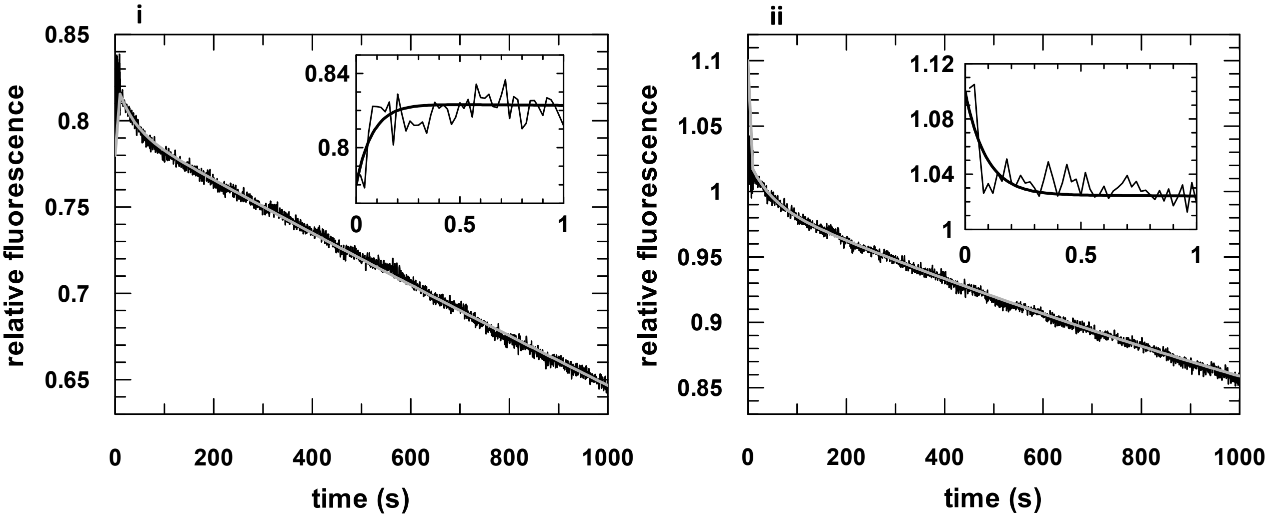

Supplementary Figure K: Dissociation of ternary hAgo2-guide-target complexes. Preassembled complexes consisting of 500 nM hAgo2, (i) 20 nM guide RNA (as2b^{FAM}) or (ii) guide DNA (D-as2b^{FAM}) and (i) 20 nM target DNA (D-s2b) or (ii) target RNA (s2b) were rapidly mixed with 2 µM unlabeled guide competitor. Representative graphs are shown. The inset shows the reaction on a shorter time scale. In both cases data could be fitted best using a triple exponential equation, yielding the following rate constants: (i) k_{-1}: 12.7 (± 1.1) s^{-1}, k_{-2}: 0.03 (± 0.01) s^{-1} and k_{-3}: 0.0004 (± 0.00001) s^{-1} and (ii) k_{-1}: 11.0 (± 0.6) s^{-1}, k_{-2}: 0.02 (± 0.0007) s^{-1} and k_{-3}: 0.0003 (± 0.00002) s^{-1}.

i

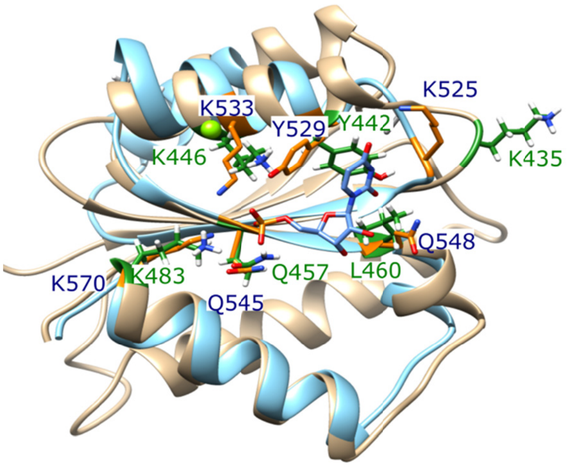

ii

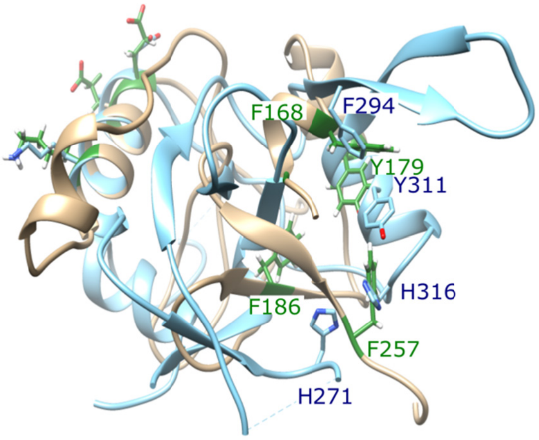

Supplementary Figure L: Comparison of the Mid and the PAZ nucleic acid binding interface of a MjAgo homology model [1] and the hAgo2 X-ray crystal structure (pdb: 4W5N). Superposition of the Mid (i) and PAZ (ii) domains of MjAgo (beige) and hAgo2 (blue). Amino acids described to be important for the binding of the 5'- and the 3'-end of the guide by hAgo2 are highlighted in orange and labelled in blue. Their corresponding residues in the MjAgo homology are depicted and labelled in green.

1. Zander A, Holzmeister P, Klose D, Tinnefeld P, Grohmann D. Single-molecule FRET supports the two-state model of Argonaute action. RNA Biol 2014;11:45–56. doi:10.4161/rna.27446.
